# Predicting students at risk of academic failure using ensemble model during pandemic in a distance learning system

**DOI:** 10.1186/s41239-021-00300-y

**Published:** 2021-12-02

**Authors:** Halit Karalar, Ceyhun Kapucu, Hüseyin Gürüler

**Affiliations:** 1 Department of Computer Education and Instructional Technologies, Faculty of Education, Muğla Sıtkı Koçman University, Muğla, Turkey; 2 Department of Informatics, Muğla Sıtkı Koçman University, Muğla, Turkey; 3 Department of Information Systems Engineering, Faculty of Technology, Muğla Sıtkı Koçman University, Muğla, Turkey

**Keywords:** Predicting student performance, Predicting student at risk, Ensemble learning model, Educational data mining, Distance learning, COVID-19 pandemic, Education in pandemic

## Abstract

Predicting students at risk of academic failure is valuable for higher education institutions to improve student performance. During the pandemic, with the transition to compulsory distance learning in higher education, it has become even more important to identify these students and make instructional interventions to avoid leaving them behind. This goal can be achieved by new data mining techniques and machine learning methods. This study took both the synchronous and asynchronous activity characteristics of students into account to identify students at risk of academic failure during the pandemic. Additionally, this study proposes an optimal ensemble model predicting students at risk using a combination of relevant machine learning algorithms. Performances of over two thousand university students were predicted with an ensemble model in terms of gender, degree, number of downloaded lecture notes and course materials, total time spent in online sessions, number of attendances, and quiz score. Asynchronous learning activities were found more determinant than synchronous ones. The proposed ensemble model made a good prediction with a specificity of 90.34%. Thus, practitioners are suggested to monitor and organize training activities accordingly.

## Introduction

After the World Health Organization declared the COVID-19 coronavirus as a global pandemic on March 11, 2020 (WHO, 2020), governments had to make strategic decisions to cope with the virus. Almost all fields of life were affected by these decisions. Educational institutions were temporarily closed in many countries. According to United Nations Educational Scientific and Cultural Organization data dated April 2, 2020 (UNESCO, 2020), approximately 1.5 billion students (about 85% globally) in 172 countries were affected by the closure of schools. In Turkey, with decisions taken by the Council of Higher Education (2020), compulsory distance education (CDE) started in universities on March 13, 2020. This decision is still valid in the first half of 2021.

For higher education institutions, predicting students at risk during the pandemic becomes more important as the students may feel isolated during CDE. To reduce this risk, it is important to give students encouraging and supportive feedback on time. Students at risk need to be anticipated first during CDE. For over ten years, researchers are trying to develop a solution by data mining (DM) and machine learning (ML) techniques that can analyze and predict students’ performance and their root cause (Injadat et al., 2020; Romero & Ventura, 2013). To the best of our knowledge, there is no study that predicts students at risk of academic failure during the pandemic.

To improve student performance and efficiency and effectiveness of higher education, prediction of students at risk of academic failure is also essential for timely instructional interventions (Adejo & Connolly, 2017; Helal et al., 2018). Studies to predict student performance have gained significant momentum in the last decade (Abu Saa et al., 2019). These predictions are mainly focused on classification and regression models. Classification (pass/fail) is more popular than the prediction of final grade or score (Khan & Ghosh, 2021; Peña-Ayala, 2014). Different classification algorithms have yielded notable results on various subjects i.e. random forest (Gray & Perkins, 2019; Kumar & Singh, 2017), fuzzy logic (Yildiz et al., 2013), k-means clustering (Sisovic et al., 2016), naive bayes (Kotsiantis et al., 2004), decision tree (Bunkar et al., 2012; Guruler et al., 2010), support vector machines (Tekin, 2014), artificial neural network (Aydoğdu, 2019), and k-nearest neighbor (Nouri et al., 2019). Differences in their achievements are quite normal as the students' data set is different. The same algorithms may show different performances for different data sets (Injadat et al., 2020; Kotsiantis et al., 2006). Moreover, each algorithm has some biases depending on the type of data it is applied to, which can make it difficult to determine the universally acceptable algorithm. Therefore, it is recommended to use ensemble learning models that combine the predictions of different algorithms to exceed the generalization capability, the robustness of a single learning algorithm and to make more accurate predictions. (Kotsiantis et al., 2010). Studies have to be conducted with modern ML algorithms where both synchronous and asynchronous learning logs obtained from large sample groups are included in the analytical process (Corsatea & Walker, 2015; Korkmaz & Correia, 2019; Romero et al., 2013).

This study mainly aimed to create and optimize an ensemble model to predict students at risk of academic failure during the pandemic. About a hundred trials were applied on different ensemble models that combine quadratic discriminant analysis (QDA), decision tree (DT), random forest (RF), extra trees (ET), logistic regression (LR), and artificial neural network (ANN) classification algorithms. In this study, answers were sought for the following questions:

- Which ensemble model is the best for predicting students at risk of academic failure during the pandemic?
- Which feature(s) of students affect the predictive performance?

This manuscript is organized as follows: background in “Background” section, methodology of the research in “Methodology” section, results in “Results” section, discussion in “Discussion” section while conclusion and suggestions are provided in “Conclusion and suggestion” section.

## Background

### Predicting student performance

DM is an information discovery process that uncovers the hidden structures in large data sets and gets meaningful information for decision makers (Romero et al., 2013). ML focuses on the design and development of algorithms that allow computers to develop behavior and generate rules based on empirical data (Singh & Lal, 2013). It automatically recognizes complex patterns based on past or current data. It predicts what will be the value of a target feature in a large data (Singh & Lal, 2013). In recent years, DM and ML algorithms are widely being used in education, finance, marketing, healthcare, engineering, and security to increase their efficiency and quality. These algorithms can be efficiently used in higher education for student pattern discovery, automation, student modeling, and academic performance prediction (Adejo, & Connolly, 2017).

During the COVID-19 pandemic, the use of learning management systems (LMS) in distance education systems has increased exponentially, which has produced big educational data. However, manual analysis of these data is not possible (Romero et al., 2008). With the application of DM or ML algorithms to facilitate the analysis of educational data, two new fields of study have emerged i.e., Educational Data Mining (EDM) and Learning Analytics (LA). EDM & LA intersects computer science, education, and statistics (Romero & Ventura, 2013, 2020). The main subjects of LA are the prediction of performance, decision support for teachers and learners, detection of behavioral patterns and learner modeling, and dropout prediction (Du et al., 2021). Benefits of LA for education include increased engagement of students, improved learning outcomes, identification of students at risk, providing real-time feedback, and personalization of learning (Banihashem et al., 2018). EDM focuses on developing models to improve the learning experience and institutional effectiveness (Dutt et al., 2017; Hussain et al., 2021). In the closely related EDM & LA (Siemens & Baker, 2012) aims to understand and optimize the learning process (Gašević et al., 2016). Therefore, the prediction of student performance has an important place in the studies conducted in these fields (Banihashem et al., 2018; Du et al., 2021; Peña-Ayala, 2014; Romero & Ventura, 2020). More specifically, predicting students at risk of failing a course (classification problem) and predicting students' final grades (regression problem) are two areas of study commonly studied.

Previous studies related to EDM & LA have successfully applied to predict students’ academic performance (Aydoğdu, 2019; Bunkar et al., 2012; Gray & Perkins, 2019; Kotsiantis et al., 2004; Kumar & Singh, 2017; Nouri et al., 2019; Sisovic et al., 2016; Tekin, 2014; Yildiz et al., 2013). According to Peña-Ayala (2014), 60% of EDM research articles have used the predictive DM approach. Similarly, Shahiri et al. (2015) reviewed predicting students’ performance using DM techniques and found that the cumulative grade point average (GPA) and internal assessments are the most frequent attributes. They also found that decision trees (DT) and artificial neural networks (ANN) were the most frequently used DM techniques for predicting students’ performance. Similarly, Abu Saa et al. (2019) reviewed and analyzed 36 research articles from 2009 to 2018 and found DTs, naïve Bayes (NB), and ANNs as the most common DM algorithms to predict and classify students’ factors. The factors affecting the student’s performance were found as students’ previous grades, class performance, e-learning activity, students’ demographics, and social information. Tomasevic et al. (2020) conducted a comprehensive analysis to compare supervised ML techniques and found ANNs as the best by feeding the student engagement data and past performance data for both classification and regression tasks. They did not find any influence of demographics on predictions.

Recent studies have tried to identify the best classification algorithm to predict student performance (Akçapınar et al., 2019; Kotsiantis et al., 2004; Nouri et al., 2019). On the other hand, Helal et al. (2018) focused on different classification algorithms to predict student performance considering student heterogeneity. A general discourse is that all works together; no individual method exhibits superior performance but rule-based algorithms, such as DTs, provided the highest interpretability. Iatrellis et al. (2021) tried to adopt a two-phase ML approach by exploiting both supervised and unsupervised learning techniques for predicting student outcomes where consolidated models produced relatively high accurate predictions.

Eventually, predicting the students’ performance has become a challenging task. Existing prediction methods are still insufficient to predict the students’ performance in higher educational institutions (Abu Saa et al., 2019). Thus, there was a clear requirement for more advanced methods predicting students at risk and determining what features affect students' outcomes; that has led us to perform this study.

### Ensemble learning

The use of ensemble learning is recommended to increase the stability of a single learning algorithm and its prediction accuracy (Dietterich, 2000). The ensemble learning model is based on a meta-algorithm that combines the same or different types of individually trained models to generates a final prediction (Kapucu & Cubukcu, 2021). Ensemble learning can be classified according to the variety of basic learning algorithms included in the created model and the way the model is created. First, ensemble learning can be heterogeneous or homogeneous according to the variety of learning algorithms included in the model. In the heterogeneous ensemble model, the same training data is applied to different learning algorithms or to the same algorithms with different parameter settings. In the homogeneous one, the original training data is divided into different sub-datasets and applied to the same learning algorithm by the number of sub-datasets (Wang et al., 2018). Second, ensemble learning can be divided to averaging methods and boosting methods. In averaging methods, learning models are created independently (Kapucu & Cubukcu, 2021). The predictions produced by these basic models are averaged to reduce the variance. In boosting methods, the original training data is divided into random subsets. The samples of the same selected basic learning algorithm are trained with these subsets. The predictions obtained are combined and a final prediction is generated. Boosting methods can be applied with any learning algorithm. However, they usually work best with powerful and complex algorithms.

Recently, researchers have utilized several ensemble models to predict students' success. Kotsiantis et al. (2010) aimed to fill the gap between the empirical prediction of student performance and the existing ML techniques in a distance education environment. They proposed an online ensemble of classifiers that combines an incremental version of NB, the 1-NN, and the WINNOW algorithms using the voting methodology. They found the proposed algorithm as the most appropriate to construct a software support tool. Injadat et al. (2020) proposed a systematic approach based on the Gini-index and p-value to select a suitable ensemble learner from a combination of various ML algorithms. They analyzed two different datasets at two separate stages of course delivery (20% and 50% respectively). Experimental results showed that the ensemble models achieve high accuracy with the low false-positive rate (FPR) at all stages for both datasets.

## Methodology

**Fig. 1:**
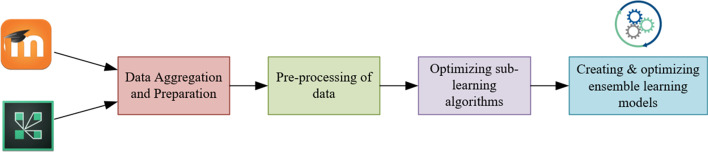
Schematic representation of the system for predicting student at risk

This study utilized an ensemble model that combines various supervised classification algorithms using the voting methodology for predicting students at risk of academic failure during the pandemic. The predictive capacity of students in course success was measured by considering both synchronous and asynchronous activity characteristics (dataset). The prediction system of students at risk consists of the following steps: data aggregation and preparation, pre-processing of data, optimizing sub-learning algorithms, and creating & optimizing ensemble learning models (Fig. 1).

All phases were performed on the Anaconda (a free OS-independent platform) distribution with Python 3.x. Libraries used in Python were Scikit-learn (ML algorithms), Pandas (to import, build, and manipulate Data Frames), NumPy (array computing), Matplotlib and Seaborn (data visualization). Scikit-learn is a big library built on NumPy, SciPy, and Matplotlib that involves simple and efficient tools for predictive data analysis. The core of NumPy is well-optimized C code. The flexibility of Python comes with the speed of compiled code.

### Data aggregation and preparation

This study was performed in a Turkish state university. The data set consists of the data of a 15-week compulsory course entitled "Information Technologies" of all 1st grade students and obtained at the end of the 2020-fall semester. This study incorporates activity data obtained from the university LMS (Moodle) and Conference Management Software (Adobe Connect) spanning a course semester. The Moodle and Adobe Connect records have different activities and resources for the students. Table 1 represents the meanings of different activity attributes. To conduct supervised experiments with these data sets, the GPA score result (pass/fail) of the course was chosen as the target column as a general preference to determine student performances. GPA score is obtained from the 40% of the midterm exam and 60% of the final exam. GPA was classified into two categories: (1) Pass: >  = 50, (2) Fail: < 50.

**Table 1:** Feature descriptions and data ranges of the dataset

Feature name	Description	Type	Value range
Gender	Gender (F/M)	Categorical	0–1
Degree	College (2-year) or faculty (4-year)	Categorical	0–1
Lecture_notes	Number of downloaded lecture notes in PDF format	Numeric	0–203
Materials	Number of other downloaded course materials	Numeric	0–176
Video	Total time spent watching recorded course videos (minutes)	Numeric	1–5988
Live	Total time spent in live course sessions (minutes)	Numeric	1–1295
Live_attendance	Total number of attendances in live course sessions	Numeric	1–46
Quiz	Quiz score	Numeric	0–100

The number of student records was 2.116. Features of the dataset consist of both categorical variables (degree, gender) and numeric variables such as the download numbers, number of attendance, and total time spent in minutes. The quiz score inherently ranges between 0 and 100 as given in Table 1.

### Pre-processing of data

Student data was extracted from Moodle and Adobe Connect, merged, and filtered according to certain criteria. Records containing inconsistent values were cleared. For example, samples which of that have a high number of live course sessions but very low attendance time were removed from the data set. As a result, the records of 2.045 student’s data set were evaluated in this study.

**Fig. 2:**
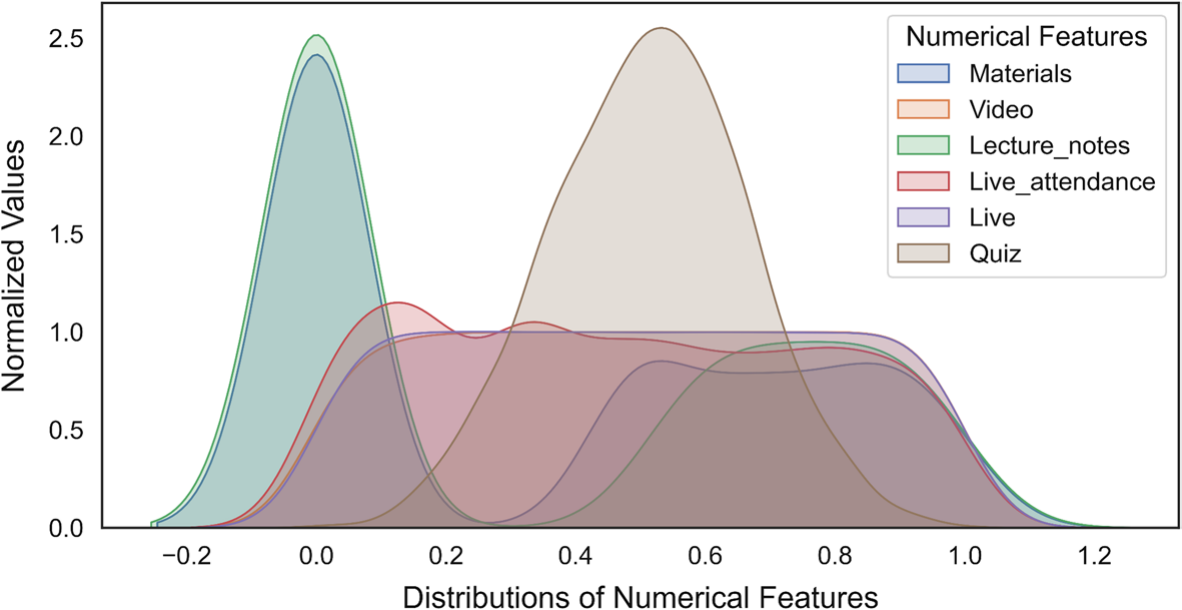
Distributions of all features

In the first column of Table 2, the features (Lecture_notes, Materials, Video, Live_attendance, and Live) that do not show the normal distribution in the data set are listed. These features were normalized using the percentage linearization method. As a result, the relevant features (Table 2) and skewness value approached zero, and got an almost normal distribution shape (Fig. 2).

**Table 2:** New skewness values for the features

Feature name	Skewness
Lecture_notes	0.3191
Materials	0.2832
Video	− 0.0094
Live_attendance	0.1109
Live	0.0015

The last thing prior to creating the ensemble model to determine student performance is feature selection. In this step, the most suitable features in the data set were determined and selected in the training of all candidate learning algorithms to create the final ensemble model. The functions in the sklearn.feature_selection module were used to select features in sample sets to enhance the predictors' accuracy scores or to increase their performance. SelectKBest, which is a univariate feature selection method, was used here. SelectKBest removes all features except k highest scoring features. Univariate feature selection works by selecting the best features according to univariate statistical tests. It can be viewed as a preprocessing step before training algorithms. Table 3 shows SelectKBest univariate statistical test scores of the features in descending order.

**Table 3:** SelectKBest test scores of the features in the student data set

Feature name	Score
Quiz	24.49
Degree	20.96
Lecture_notes	13.92
Materials	9.30
Video	1.60
Gender	1.53
Live_attendance	0.48
Live	0.15

It is clear from Table 3 that some features (such as Quiz) are effective in predicting student performance. This is an expected observation; there was no unexpected change in this regard during the pandemic. However, the effects of video, live_attendance and live features, which are the features of synchronous education, on students' performance are surprisingly low. Gender, on the other hand, was not seen as a relevant factor in course success. Features other than those whose impact factors are very low according to the statistical test score value (Gender, Live_attendance, and Live) were selected to train the model.

### Optimizing sub-learning algorithms

This study used an ensemble learning model involves Gradient Boosting (GB), Quadratic Discriminant Analysis (QDA), Decision Tree (DT), Random Forest (RF), Extra Trees (ET), Logistic Regression (LR), and Artificial Neural Network (ANN) classifiers as candidate algorithms. In algorithm set selection, the classification generally gave successful results where each one looked at the classification problem from a different perspective. The general features of the selected algorithms are discussed in the following lines.

GB is based on the intuition that the best possible next model, when combined with previous models, minimizes the overall prediction error. The basic idea is to set target results for this next model to minimize error. GB can be used for both classification and regression. QDA (McLachlan, 1992) is a classification algorithm based on the statistical discrimination method that uses a quadratic decision surface to separate samples of two or more classes of objects or events. RF (Breiman, 2001) and ET (Geurts et al., 2006) models are specialized forms of the bagging method where they use only DT as the base learning algorithm. In bagging methods, the original training data is divided into random subsets. Identical copies of the selected basic learning algorithms are trained with these subsets. A combination of these predictions is used to obtain a final prediction. These methods are used to reduce the variance of the underlying algorithm. Thus, overfitting, which is a problem of classification, is also reduced. In RF and ET models, there are multiple DTs. They give the averaged prediction of these combined trees. In contrast to the RF, ET model uses a complete training dataset rather than re-sampled replicas to grow the trees and split the tree nodes randomly (Kapucu & Cubukcu, 2021). LR is a statistical model that employs a logistic function to model a binary dependent variable. A binary logistic model has a dependent variable mathematically with two potential values i.e., pass/fail, indicated by 0 and 1. ANN is a parallel and distributed information processing structure inspired by the human brain, consisting of interconnected processing units (Russell & Norving, 2020). This structure is made up of a combination of artificial nerve cells—neurons—created by mimicking the functions of biological nerve cells. Artificial neurons are entirely nature-inspired units and information systems that can imitate the human brain's learning capacity. A neural network is a network structure created by the attachment of neurons to one another (Haykin, 2008).

While determining whether a learning algorithm is suitable for the field and data used, the model obtained with the training of the algorithm is tested against a dataset containing previously not encountered examples. During testing a model, the data source remains the same, while the samples must be new. For this reason, it is the most preferred method to divide the owned dataset into training and testing parts. In cases of the scarcity of samples, the generalization success in the data may decrease due to the incomplete training of the learning algorithm. Thus, the training/test split method is more suitable for large data sets. To overcome this problem, cross validation (CV) method was proposed (Allen, 1974; Geisser, 1975). The CV is a training-test split method dividing the original dataset into the (K) pieces. Each piece is used in both training and testing parts of the created model.

Grid-search function was used to optimize candidate algorithms. This function performs an iterative search to find the optimal hyperparameter values for a particular learning algorithm. Using CV during the search process, the function reports the best candidate parameters and the prediction accuracies obtained with these settings as a result. While optimizing the model within the scope of the research, tenfold CV for the hyperparameters (Table 4) and grid-search was carried out by observing the specificity performance.

**Table 4:** Grid-search parameters for each candidate algorithm

Algorithms	Hyperparameter space to improve algorithm performance
DT	Measure of impurity = ['gini', 'entropy'] & Split strategy = ['best', 'random'] & Max depth = [None, 3] & Max features = ['auto', 'sqrt', 'log2'] & Class weight = ['balanced', None]
RF	Measure of impurity = ['gini', 'entropy'] & Bootstrap = [False, True] & Max depth = [None, 3] & Warm start = [False, True] & Class weight = ['balanced', 'balanced_subsample'] & Number of trees = [100, 200, 300, 400]
ET	Measure of impurity = ['gini', 'entropy'] & Max depth = [None, 3] & Warm start = [False, True] & Class weight = ['balanced', 'balanced_subsample'] & Number of trees = [100, 200, 300, 400]
LR	Optimization algorithm = ['newton-cg', 'lbfgs', 'liblinear', 'sag', 'saga'] & Inverse of regularization strength = [0.1, 1, 10, 100] & Class weight = ['balanced', None] & Dual = [False, True] & Fit intercept = [False, True] & Tol = [0.001, 0.01] & Warm start = [False, True]
GB	Measure of impurity = ['friedman_mse', 'mse', 'mae'] & Max depth = [None, 3] & Number of trees = [100, 200, 300]
ANN	Alpha = [0.1, 0.01, 0.001] & Activation = [‘relu’, ‘logistic’] & Early stopping = [True, False] & Number of hidden layers = [200, 300, 400]
QDA	*No parameter settings available in Scikit-learn implementation

Decision Tree (DT), Random Forest (RF), Extra Trees (ET), Logistic Regression (LR), Gradient Boosting (GB), Artificial Neural Network (ANN), and Quadratic Discriminant Analysis (QDA)

**Fig. 3:**
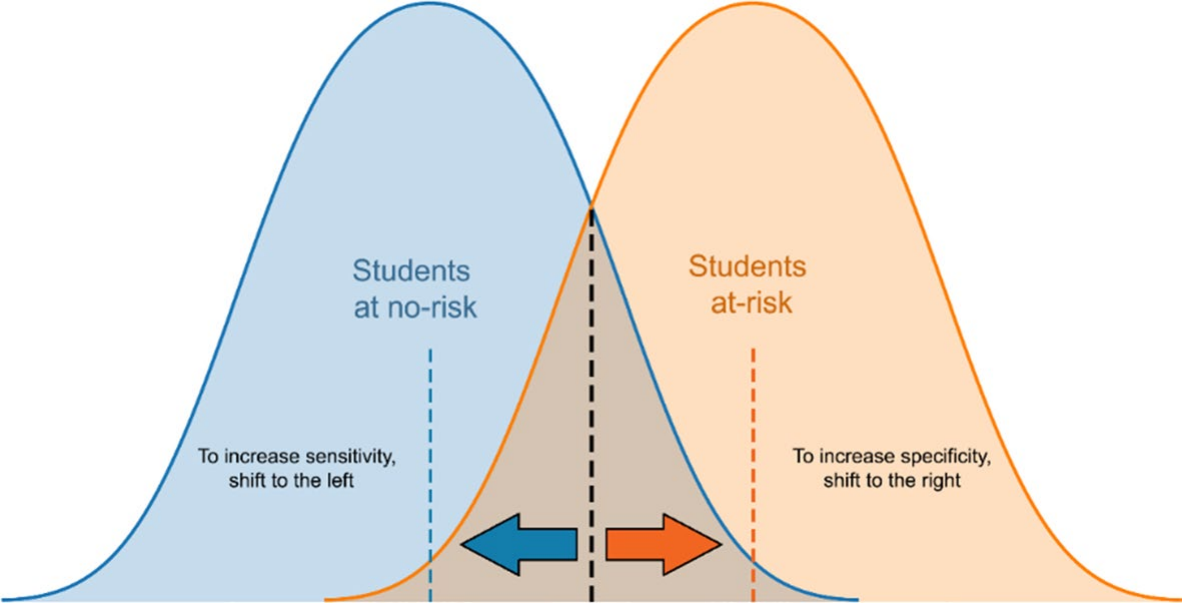
Sensitivity specificity trade-off. It is adapted from Steward (2019)

Specificity performance metric can make a classification in which students with academic risks can be predicted more accurately, which is the main purpose, without ignoring the successful students (Fig. 3). By shifting to the right, the proportion of FPs decreases, which means improved specificity.

This stated performance measure is commonly called as true negative rate (TNR) and selectivity. A model tested in this way achieves a high TN result with a low FP to get a high score. The Scikit-learn version of the QDA algorithm was used with its default settings since there are no hyperparameters that can be adjusted for performance. The metrics that are preferred and frequently used in the literature while measuring the classification performance of the model are briefly introduced below. These metrics use true positive (TP), true negative (TN), false positive (FP), and false negative (FN) values derived from the confusion matrix (Table 5).

**Table 5:** Classification performance metrics

Metric	Explanation	Formula
Accuracy	How often are the model's predictions correct?	(TP + TN) / (TP + TN + FN + FP)
Precision	When the model predicts positive, how often is it correct?	TP / (TP + FP)
Sensitivity (Recall) or TPR	When it is actually positive, how often it correctly predicts?	TP / (TP + FN)
Specificity or TNR	When it is actually negative, how often it correctly predicts?	TN / (TN + FP)
Balanced accuracy	The mean of sensitivity and specificity	(TPR + TNR) / 2
F-Measure	The harmonic mean of precision and sensitivity	2 × Precision × Recall / (Precision + Recall)

### Creating and optimizing ensemble learning models

To find the most successful ensemble model, first, all learning algorithms are combined in double, triple, and quadruple sets. In this way, the created models try to better predict the target class by conceptually combining different sub-learning models. Within the scope of this study, candidate ensemble models were created in which each learning model in the group has equal weight and equal voting rights (Voting Classifier). Therefore, all ensemble models created in this step were tested using tenfold CV with balanced accuracy performance metric. In the ranking obtained, the most successful candidate ensemble models were determined. This metric ensures that among all candidate ensemble models, especially those who can predict both target classes with high accuracy, are at the top.

Finally, after finding the most successful candidate ensemble models, they were optimized. For this, and to determine the best of them as the final ensemble model, an iterative search was made in the different voting types and voting rights weights listed in Table 6. In this search process, the most successful candidate ensemble models were tested with these different settings using a specificity performance metric with tenfold CV.

**Table 6:** Optimization settings of the most successful candidate ensemble models

Parameters	Options
Voting type	‘soft’, ‘hard’
Weights	None, [1,1,2], [1,2,1], [2,1,1], [1,2,2], [2,2,1], [2,1,2]

## Results

### Optimizing sub-learning algorithms

The test statistics of the learning algorithms used in the study are given in Table 7. Accordingly, the most successful models in the TN results, which show the correct predictions of unsuccessful (fail) students in the test dataset, were RF, LR, and DT, while the most unsuccessful models were QDA, ANN, and GB. ET model was moderately successful.

**Table 7:** Test statistics of the individual algorithms optimized with grid-search

Models	TN	FP	FN	TP
Gradient Boosting (GB)	91	54	38	431
Quadratic Discriminant Analysis (QDA)	81	64	25	444
Extra Trees (ET)	112	33	107	362
Decision Tree (DT)	120	25	250	219
Random Forest (RF)	125	20	100	369
Logistic Regression (LR)	121	24	89	380
Artificial Neural Network (ANN)	86	59	26	443

True positives (TP), True negatives (TN), False positives (FP), and False negatives (FN)

Table 8 shows the calculated performance scores in the range of [0–1] based on the prediction results obtained by the models on the test dataset. The most successful models in the specificity values showing the correct estimation rates of the failed students in the dataset were RF, LR and DT, while the most unsuccessful models were QDA, ANN and GB. ET model was moderately successful. In the sensitivity values, which show the correct estimation rates of the students who passed the course in the dataset, the most successful models were QDA, ANN and GB, while other models exhibited lower success.

**Table 8:** Performance scores of the individual algorithms optimized with grid-search

	GB	QDA	ET	DT	RF	LR	ANN
Specificity or TNR	0.6276	0.5586	0.7724	0.8276	*0.8621*	0.8345	0.5931
Sensitivity or TPR	0.9190	*0.9467*	0.7719	0.4670	0.7868	0.8102	0.9446
Precision	0.8887	0.8740	0.9165	0.8975	*0.9486*	0.9406	0.8825

Italic characters show most successful scores of each row

Gradient Boosting (GB), Quadratic Discriminant Analysis (QDA), Extra Trees (ET), Decision Tree (DT), Random Forest (RF), Logistic Regression (LR), and Artificial Neural Network (ANN)

### Creating and optimizing ensemble learning model

A total of 91 candidate ensemble models were created and tested using double, triple and quadruple combinations from seven classification algorithms. Among these models, test statistics of the 5 most successful ensemble models that can predict both target classes (passing or failing) together with high accuracy by using balanced accuracy performance metric are given in Table 9.

**Table 9:** Test statistics of the top five candidate ensemble models with optimization

Candidate ensemble and its sub-models	TN	FP	FN	TP
ET + RF + LR	*131*	14	110	359
QDA + LR	121	24	89	380
GB + ET + RF + LR	121	24	91	378
GB + DT + LR	117	28	90	379
GB + DT + LR + ANN	118	27	91	378

Italic characters show highest true negative prediction.

Gradient Boosting (GB), Quadratic Discriminant Analysis (QDA), Extra Trees (ET), Decision Tree (DT), Random Forest (RF), Logistic Regression (LR), and Artificial Neural Network (ANN)

**Fig. 4:**
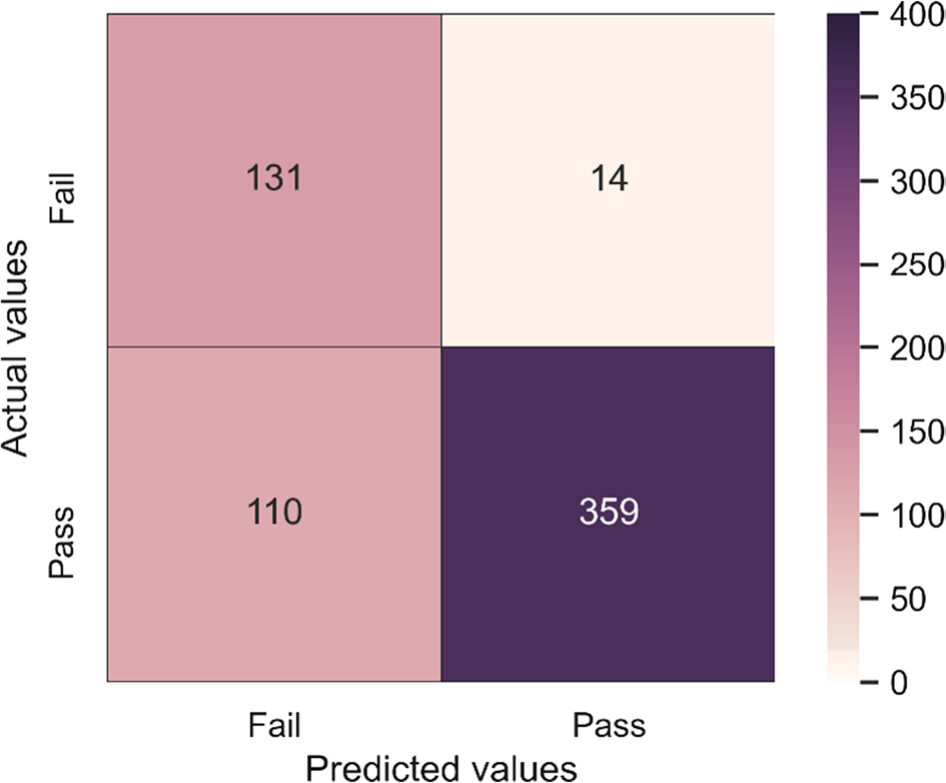
Confusion matrix of the final ensemble model

At the end of search process, the 1st candidate model formed by ET, RF, LR sub-models was selected as the final ensemble model. In the final ensemble model, "hard" voting type and [1,2,1] model weights were determined as the best parameters for optimization. Figure 4 shows the error matrix of the selected final ensemble model.

First three rows of Table 10 show the performance scores of all models on the test dataset. In the last row of the table, the average of the estimation accuracies performed on the whole dataset with tenfold CV, considering the specificity performance metric of all models, is given. Italic characters show most successful scores of each row. Since this study aimed to predict students at risk, the specificity-TNR (first row) values shown in Table 10 are more important. The final ensemble model achieved the highest score both on the test dataset only and on the whole dataset with CV according to the specificity performance metric. At the same time, it was the model that achieved the highest score in the precision performance metric.

**Table 10:** Performance scores of individual algorithms and ensemble model

	GB	QDA	ET	DT	RF	LR	ANN	ELM
Specificity	0.6276	0.5586	0.7724	0.8276	0.8621	0.8345	0.5931	*0.9034*
Sensitivity	0.9190	*0.9467*	0.7719	0.4670	0.7868	0.8102	0.9446	0.7655
Precision	0.8887	0.8740	0.9165	0.8975	0.9486	0.9406	0.8825	*0.9625*
CV—Specificity	0.6099	0.5768	0.7841	0.8445	0.8425	0.8362	0.5768	*0.8861*

Gradient Boosting (GB), Quadratic Discriminant Analysis (QDA), Extra Trees (ET), Decision Tree (DT), Random Forest (RF), Logistic Regression (LR), Artificial Neural Network (ANN), Ensemble Learning Model (ELM)

**Fig. 5:**
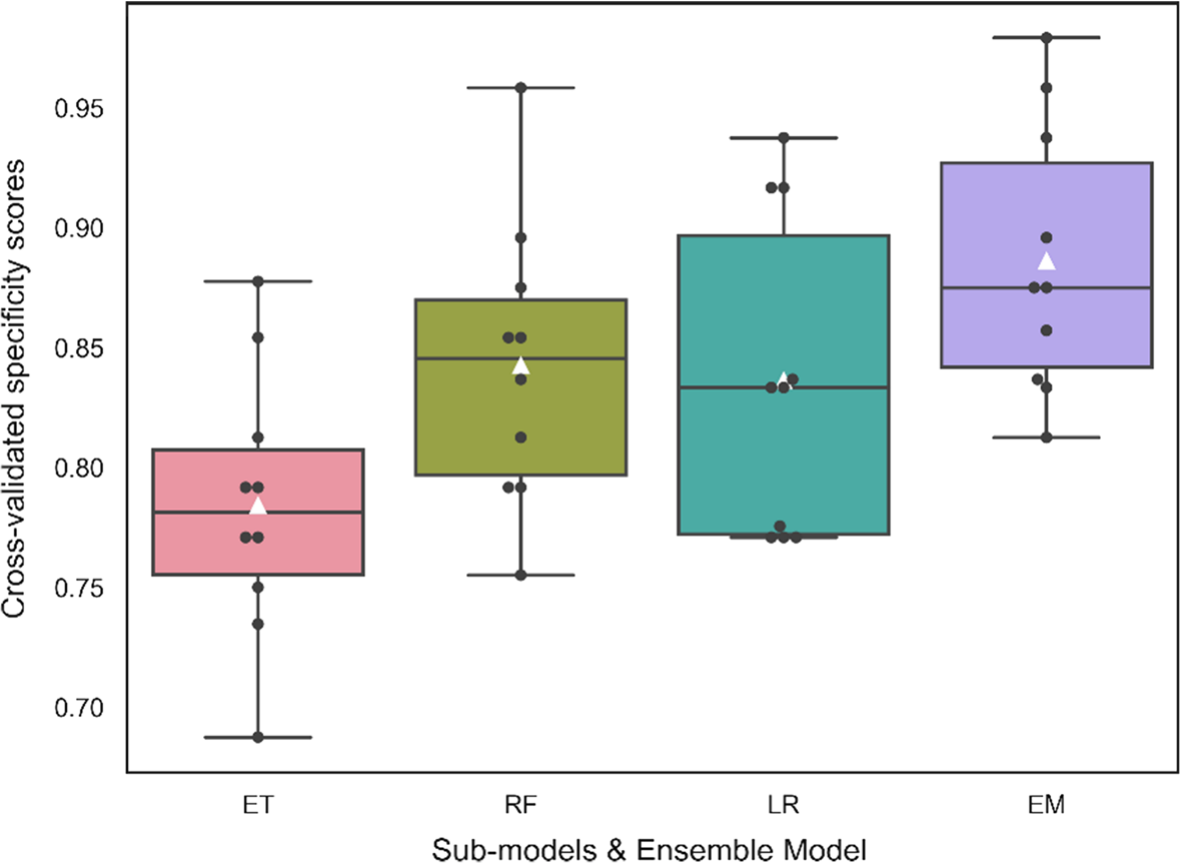
Comparison of individual sub-models and ensemble model

Figure 5 shows the cross-validated specificity scores of individual algorithms and ensemble model. The box-plot visual comparison of the specificity scores for ET, RF, LR sub-models and optimized ensemble model was obtained with tenfold CV. The black dots on each box-plot represent the scores obtained at each fold of the CV, while the white triangles represent the average of these scores. As can be seen, the average score obtained by the final ensemble model on the far right of the graph is higher than all of the three sub-models.

## Discussion

The question is, which ensemble model is the best for predicting students at risk of academic failure during the pandemic? To answer this question, 91 candidate ensemble models were created and tested with double, triple and quadruple combinations from classification algorithms using Gradient Boosting, Quadratic Discriminant Analysis, Decision Tree, Random Forest, Extra Trees, Logistic Regression, and Artificial Neural Network. The results showed that the ensemble model, which consists of combinations of Extra Trees (ET), Random Forest (RF) and Logistic Regression (LR) classification algorithms, is the best to predict students at risk after optimization. The specificity of this model, namely the TNR score is 90.34%. In previous studies, best algorithms used to predict student performance are Random Forest (Gray & Perkins, 2019; Kumar & Singh, 2017), Fuzzy Logic (Yildiz et al., 2013), K-means Clustering (Sisovic et al., 2016), Naive Bayes (Kotsiantis et al., 2004), Decision Tree (Bunkar et al., 2012), Support Vector Machines (Tekin, 2014), Artificial Neural Network (Aydoğdu, 2019), and kNN (Nouri et al., 2019). However, this study revealed the ensemble model that gives the best prediction result with the combination of different classification algorithms instead of determining the individual algorithm that best predicts student performance. In a similar study by Kotsiantis et al. (2010), it was found that the ensemble model consisting of the combination of Naive Bayes, 1-NN, and WINNOW algorithms best predicted student performance. The results show that the ensemble models can be more successful in predicting student performance, especially in predicting students at risk. For this reason, it is recommended to use the ensemble learning approach in studies to be conducted for the prediction of student performances.

Another question is which features of students affect the predictive performance? Regarding this question, results showed that the most important factors affecting prediction performance are quiz score, degree, number of lecture notes download, number of other course materials downloaded, and total time spent watching recorded course videos. The effectiveness of student characteristics (such as quiz score and degree) used in this research in predicting student performance is obvious. This is an expected observation. The pandemic effect has not been seen here. Studies show that there is a relationship between Moodle use and student performance (Corsatea & Walker, 2015). However, findings regarding which types of interaction affect students' performance are contradictory. It shows that a multiple regression model can predict 52% of the variance in the final exam scores of the students by sending and reading messages, contributing to content production, the number of completed quizzes and examined files (Zacharis, 2015). In another study (Aydoğdu, 2019), it was concluded that the number of attendances in the live lessons, the number of attendances in the archived lessons and the time spent on the content were more effective than other variables in predicting students' performance. In another study (You, 2016), it was determined that late submission of assignments, the number of attending sessions or logging in to the course and reading the information package of the course are important variables in predicting the success of the course. As a result, the use of Moodle has a determining effect on students' performance. However, which interaction types are more effective in predicting performance here may vary depending on the context.

In this research, it is surprising that the features live (total time spent in live course sessions) and live_attendance (total number of attendances in the live course sessions) obtained are ineffective. In parallel with this finding, the scores of the students, who have attended live sessions, were not different from those who have watched the recorded videos (Nieuwoudt, 2020). Thus, the synchronous online sessions are not effective in predicting student’s performance (Gašević et al., 2016). A previous report also showed similar success of both group students who have learnt synchronously and asynchronously (Olson & McCracken, 2015). On the contrary, in another study, live course sessions were found significant in predicting students' performance (Aydoğdu, 2019; You, 2016). The strategic decisions taken by the university administration may have affected the student preferences in the current study. The students were not required to attend the live sessions, considering the possibility that some students may have no access to computer or internet connection. It was enough for them to watch the recorded videos of the lessons later at their ease. So, most of the students have watched the lessons asynchronously. On the other hand, most of the students may have lost their interest in the live lessons. In short, the live course sessions were found ineffective, however, recorded videos of the sessions were effective in predicting students at risk. Based on these findings, education administrators and policymakers should consider the use of free conference management software to efficiently use the limited resources. To increase the success of the students, it is recommended that the educators record the live sessions and share these recordings with the students.

In this study, results were obtained with five features in the training data set. It is necessary to improve the generalization ability of the established model for higher prediction accuracy. For this reason, it is recommended to add new effective features to train the model in similar studies. Also, the number of observations can be increased in order to eliminate the dependence on student, instructor, and course-specific behaviors in the data set. In addition, using data obtained from more than one semester and re-training the model multiple times within a period to increase the generalization ability of the model and thus to make a successful prediction for the next semester may increase the prediction performance. Nevertheless, a set of features obtained from the learning management system or conference management software may have a significant impact on student achievement in a specific course while not so beneficial in another (Gašević et al., 2016).

As it is known, the success of distance education largely depends on the quality of communication and interaction with the student. Therefore, administrators and policy makers should encourage instructors to use synchronous and asynchronous learning environments effectively. Instructors should also encourage students to effectively use these educational environments. Then, by increasing the generalization ability of the models trained with data containing many effective features based on communication and interaction from the bottom up, policy makers can make data-based decisions that can increase the quality of higher education.

To wind up, this study develops an ensemble model and tests on data obtained from over two thousand students instead of small groups and identifies important predictors of students at risk of academic failure during the pandemic.

## Conclusion and suggestions

Ensemble learning model provides high accuracy in many areas. Its use as a new approach is spreading rapidly in education. This study concluded that the ensemble learning model approach, which consists of combinations of Extra Trees (ET), Random Forest (RF) and Logistic Regression (LR) classification algorithms, is the best to predict students at risk of academic failure. The approach proposed in this study will help administrators, instructors, and policymakers to develop new policies and instructional interventions in higher education, as it predicts students at risk with high accuracy. With these policies and instructional interventions to be determined, students at risk can be provided with the necessary support and feedback to prevent them from falling behind or failing. This situation can increase the quality, efficiency and effectiveness of education in higher education.

Another result of the study is that quiz score, degree, number of lecture notes downloads, number of other course materials downloads, and total time spent watching recorded course videos are effective in predicting students at risk. To predict students at risk more accurately, it is recommended that instructors and instructional designers pay attention to quizzes, students' degree features, shared lecture notes, and recorded course videos while designing course content. On the other hand, synchronous learning during the pandemic was ineffective in predicting students at risk. The reasons for this situation can be investigated in depth. Features such as chat, wiki, forum, dictionary and questionnaires in the Moodle system, the verbal participation of the students in the Adobe Connect system, and the chat message records of the students with the instructor and other students during the lesson were not included in the study. In subsequent studies, it is suggested to test the ensemble learning models created with the combination of different algorithms using more data on student characteristics, and synchronous & asynchronous learning activities. More specifically, models must be trained by adding new features without letting an over-fitted model to improve the model's goodness.

## Data Availability

The data that support the findings of this study are available on request.
